# Barriers and facilitators to screening and treating malnutrition in older adults living in the community: a mixed-methods synthesis

**DOI:** 10.1186/s12875-019-0983-y

**Published:** 2019-07-15

**Authors:** Philine S. Harris, Liz Payne, Leanne Morrison, Sue M. Green, Daniela Ghio, Claire Hallett, Emma L. Parsons, Paul Aveyard, Helen C. Roberts, Michelle Sutcliffe, Siân Robinson, Joanna Slodkowska-Barabasz, Paul S. Little, Michael A. Stroud, Lucy Yardley

**Affiliations:** 1 Centre for Clinical and Community Applications of Health Psychology (CCCAHP), University of Southampton, Building 44, Highfield Campus, Southampton, SO17 1BJ UK; 2 Primary Care and Population Sciences, Aldermoor Health Centre, Aldermoor Close, Southampton, SO16 5ST UK; 3 Bournemouth University, Bournemouth House B236, 19 Christchurch Road, Bournemouth, BH1 3LH UK; 4 Friarsgate Surgery, Stockbridge Road, Winchester, SO22 6EL UK; 5 Wessex Academic Health Science Network and NIHR Southampton Biomedical Research Centre, University Hospital Southampton NHS Foundation Trust, Southampton, SO16 6YD UK; 6 Nuffield Department of Primary Care Health Sciences, Radcliffe Observatory Quarter, University of Oxford, Woodstock Road, Oxford, OX2 6GG UK; 7 Academic Geriatric Medicine, Level E Centre Block, Mailpoint 807, University Hospital Southampton, Southampton, SO16 6YD UK; 8 Community Dietetic Department, Southampton NHS Treatment Centre, Royal South Hampshire Hospital, Brintons Terrace, Southampton, SO14 0YG UK; 9 AGE Research Group, Biomedical Research Building, Campus for Ageing and Vitality, Newcastle University, Newcastle upon Tyne, NE4 5PL UK; 10 Gastroenterology and Clinical Nutrition, University Hospital Southampton NHS Foundation Trust, Tremona Road, Southampton, SO16 6YD UK; 11 Centre for Academic Primary Care and School of Psychological Science, University of Bristol, 39 Whatley Road, Bristol, BS8 2PS UK

**Keywords:** Primary health care, General practice, Malnutrition, Independent living, Health services for the aged, Dietary supplements

## Abstract

**Background:**

Malnutrition (specifically undernutrition) in older, community-dwelling adults reduces well-being and predisposes to disease. Implementation of screen-and-treat policies could help to systematically detect and treat at-risk and malnourished patients. We aimed to identify barriers and facilitators to implementing malnutrition screen and treat policies in primary/community care, which barriers have been addressed and which facilitators have been successfully incorporated in existing interventions.

**Method:**

A data-base search was conducted using MEDLINE, Embase, PsycINFO, DARE, CINAHL, Cochrane Central and Cochrane Database of Systematic Reviews from 2012 to June 2016 to identify relevant qualitative and quantitative literature from primary/community care. Studies were included if participants were older, community-dwelling adults (65+) or healthcare professionals who would screen and treat such patients. Barriers and facilitators were extracted and mapped onto intervention features to determine whether these had addressed barriers.

**Results:**

Of a total of 2182 studies identified, 21 were included (6 qualitative, 12 quantitative and 3 mixed; 14 studies targeting patients and 7 targeting healthcare professionals). Facilitators addressing a wide range of barriers were identified, yet few interventions addressed psychosocial barriers to screen-and-treat policies for patients, such as loneliness and reluctance to be screened, or healthcare professionals’ reservations about prescribing oral nutritional supplements.

**Conclusion:**

The studies reviewed identified several barriers and facilitators and addressed some of these in intervention design, although a prominent gap appeared to be psychosocial barriers. No single included study addressed all barriers or made use of all facilitators, although this appears to be possible. Interventions aiming to implement screen-and-treat approaches to malnutrition in primary care should consider barriers that both patients and healthcare professionals may face.

**Review registrations:**

PROSPERO: CRD42017071398. The review protocol was registered retrospectively.

**Electronic supplementary material:**

The online version of this article (10.1186/s12875-019-0983-y) contains supplementary material, which is available to authorized users.

## Background

Malnutrition (specifically undernutrition) can impair wound healing, reduce muscle strength and weaken the immune response, increasing many health risks including infections and delayed recovery from illness [1]. Increased prevalence of long-term health conditions makes older adults particularly vulnerable to malnutrition [2, 3]. Malnutrition can have medical or physiological causes (e.g. difficulties chewing or swallowing), psychosocial (e.g. poverty or depression [2]), or a combination of these.

In the UK, more than 3 million people are believed to be malnourished [4], and the cost associated with malnutrition across health and social care was estimated to be £20 billion in 2015 [5]. Among community-dwelling older adults in the UK and Ireland, 14% may be at risk of malnutrition [6], though estimates vary depending on the specific sub-groups and screening tools studied [7]. The terms malnutrition and undernutrition are commonly used to define the same state, which can arise through inadequate intake of nutrients or an inability of the body to make use of nutrients [8]. However, *risk* of malnutrition is sometimes conceptualised as increasing over time for as long as undernutrition continues [7]. The Global Leadership Initiative on Malnutrition (GLIM) recently agreed diagnostic criteria for malnutrition, which include meeting at least one of the following criteria (non-volitional weight loss, low body mass or low muscle strength) and additionally at least one of the following criteria (reduced food intake or assimilation or disease burden or inflammation) [8].

Treating malnutrition in older adults may improve their health, quality of life [9, 10] and reduce healthcare costs [5]. In the hospital setting, malnutrition-screen-and-treat policies are recommended [11], but there is little evidence for their implementation and value in primary care. Systematic screening, using validated tools such as the Malnutrition Universal Screening Tool [12], improves identification of individuals who may be at risk of malnutrition [4] allowing treatment which may prevent malnutrition and its consequences [13]. Treatment includes providing dietary advice [14], meals [15] or oral nutritional supplements (ONS [16]). Treatment may differ depending on the severity of malnutrition risk, and several care pathways, including for the community [17], have been developed. Care pathways include tools to aid diagnosis of underlying diseases or conditions that make eating or digestion difficult, so that these can be treated [18]. However, malnutrition remains under-recognised and untreated across all healthcare settings [19] because healthcare professionals (HCP) often fail to diagnose it [20] or attach low priority to nutrition in older patients [21]. Clinical guidelines recommend that screening should be carried out by HCPs who have received appropriate training [11, 22], but do not specify how screening should be enacted or the training delivered despite urgent calls to improve HCPs’ nutrition education [23]. Uncertainty remains about which of various approaches are most practicable and acceptable to HCPs and older adults [24]. Further, the evidence in support of systematic use of screening tools [25] and treatment approaches such as giving ONS [16] has largely emerged from research in secondary care, and comparatively little is known about how this translates to those living at home.

More research on the barriers to nutritional screening and treatment in older, community-dwelling adults [24, 26] has been called for. Previous reviews have focused on patient [27] or HCP barriers [13, 28] in isolation, or on the effectiveness of randomised controlled trials (RCTs) [24]. Given the limited evidence available [26], the current synthesis seeks to extend the literature by reviewing findings about older patients and HCPs, from both qualitative and quantitative studies, including non-RCT studies, which can, if well designed, be considered strong evidence [26] and can inform us of the acceptability and feasibility of intervention features. The core analysis, and novel contribution to the literature, is a mapping [29] of barriers, facilitators and intervention features to identify how the content and design of interventions can be optimised and to identify gaps in recent intervention research.

The aims of this synthesis are to: 1) identify barriers and facilitators to implementing malnutrition screen and treat policies in primary/community care; 2) map barriers and facilitators to features in existing interventions; and 3) make recommendations for the design of interventions targeting malnutrition in older adults and nutrition education for HCPs.

## Methods

Barriers and facilitators to screen-and-treat approaches were extracted [30] and mapped onto intervention features [29] to determine whether barriers had been addressed and what solutions were available and feasible. A meta-analytic, causal approach to the quantitative studies was considered, but deemed unsuitable because of the heterogeneity of the interventions. Instead, we used thematic synthesis and aspects of Intervention Component Analysis [30, 31] to describe and critically interpret the findings (see [30]. The protocol can be found here: http://www.crd.york.ac.uk/PROSPERO/display_record.php?ID=CRD42017071398 (PROSPERO registration number CRD42017071398).

### Literature search

Seven databases (MEDLINE, Embase, PsycINFO, DARE, CINAHL, Cochrane Central and Cochrane Reviews) were searched in June 2016. Search terms are shown in Additional file 1. The search was restricted to references from 2012 onwards, to focus on publications since Cochrane reviews on malnutrition screening [32] and interventions for malnutrition [33]. LP, DG and JS screened titles and abstracts and excluded irrelevant references. LP and PH screened full text publications for eligibility. Qualitative and quantitative intervention studies and studies exploring older people’s eating patterns or appetite or health professionals’ experiences in relation to undernutrition were included if participants were either adults 65+ living at home or healthcare professionals who would care for these participants. Studies were excluded if participants were care home residents or hospital inpatients, or if participants presented with a terminal disease, cancer, dementia or diabetes, who may have specific nutritional requirements due to their conditions. Studies were also excluded if they were not in English. Inclusion/exclusion criteria are shown in Additional file 2.

### Data coding, extraction and synthesis

**Fig. 1:**
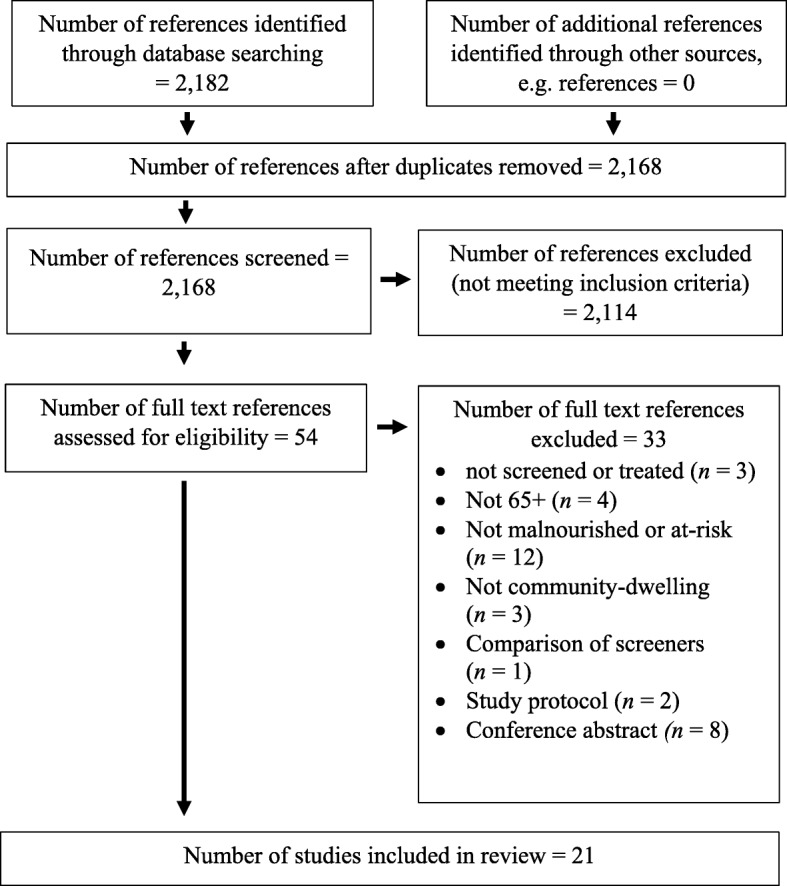
Flow chart of studies included in the synthesis

Key study characteristics were extracted and tabulated (Additional file 4: Tables S4-S5). Figure 1 is a flow chart outlining eligible studies containing qualitative and quantitative data; those presenting primarily quantitative data will be referred to as “interventions” and included RCTs (*n* = 6), RCT feasibility (*n* = 3) and pre-post designs (*n* = 4).

Papers reporting on the studies (all sections bar the introduction, following Corbett and colleagues [30]) were coded line-by-line and codes organised into descriptive themes, in line with thematic synthesis [34]: PH and LP established an initial coding manual with the aim of capturing barriers and facilitators to malnutrition-screen-and-treat approaches and intervention features designed to address barriers and incorporate facilitators. PH and LP double-coded a subset of studies (8 of 21) using this coding manual. Discrepancies were discussed and the coding manual was refined accordingly. PH coded the remaining studies. LP read all remaining studies and resulting codes, and the findings and additional codes were discussed with all authors. The emerging codes were organised into barriers and facilitators, for patients and HCPs, to screening, nutritional self-care and ONS use.

Following Shepherd and colleagues [29], the resulting data were first analysed and synthesised narratively to provide an overview of included studies. Syntheses are not reported here; findings are similar to previous reviews, e.g. [24, 28] Then, novel to malnutrition screening literature and reported here, intervention and qualitative studies were synthesised to map barriers and facilitators onto intervention features in a matrix, identifying which interventions (if any) had addressed barriers or incorporated facilitators. Of note, in some instances no facilitator was explicitly named in the reviewed studies, but a possible solution to addressing the barrier was found in intervention features. All authors read and commented on the draft synthesis and provided clinical and / or nutritional expertise during search strategy development and analysis of findings.

**Fig. 2:**
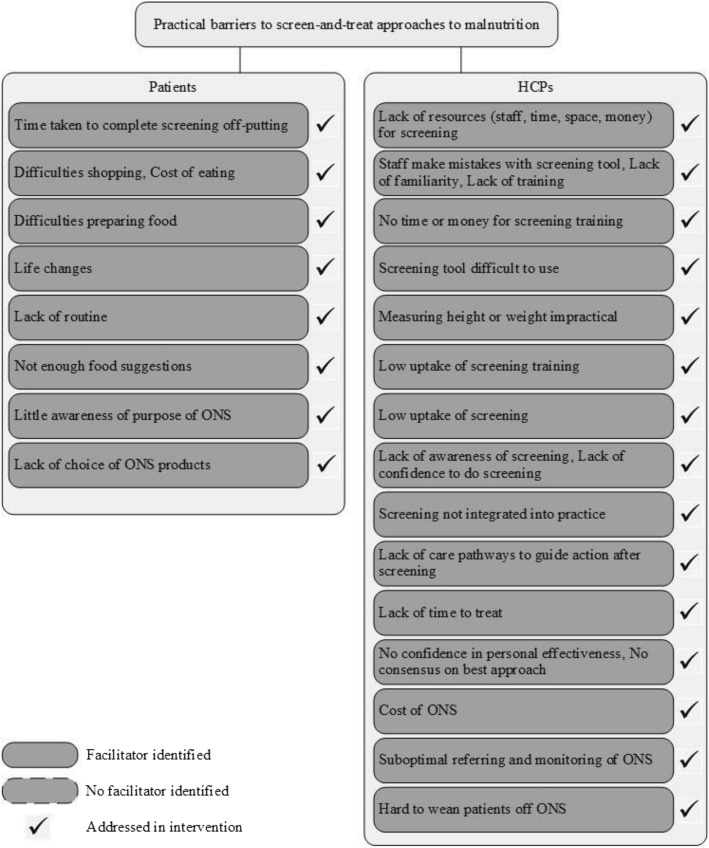
Practical barriers to screen-and-treat approaches to malnutrition

**Fig. 3:**
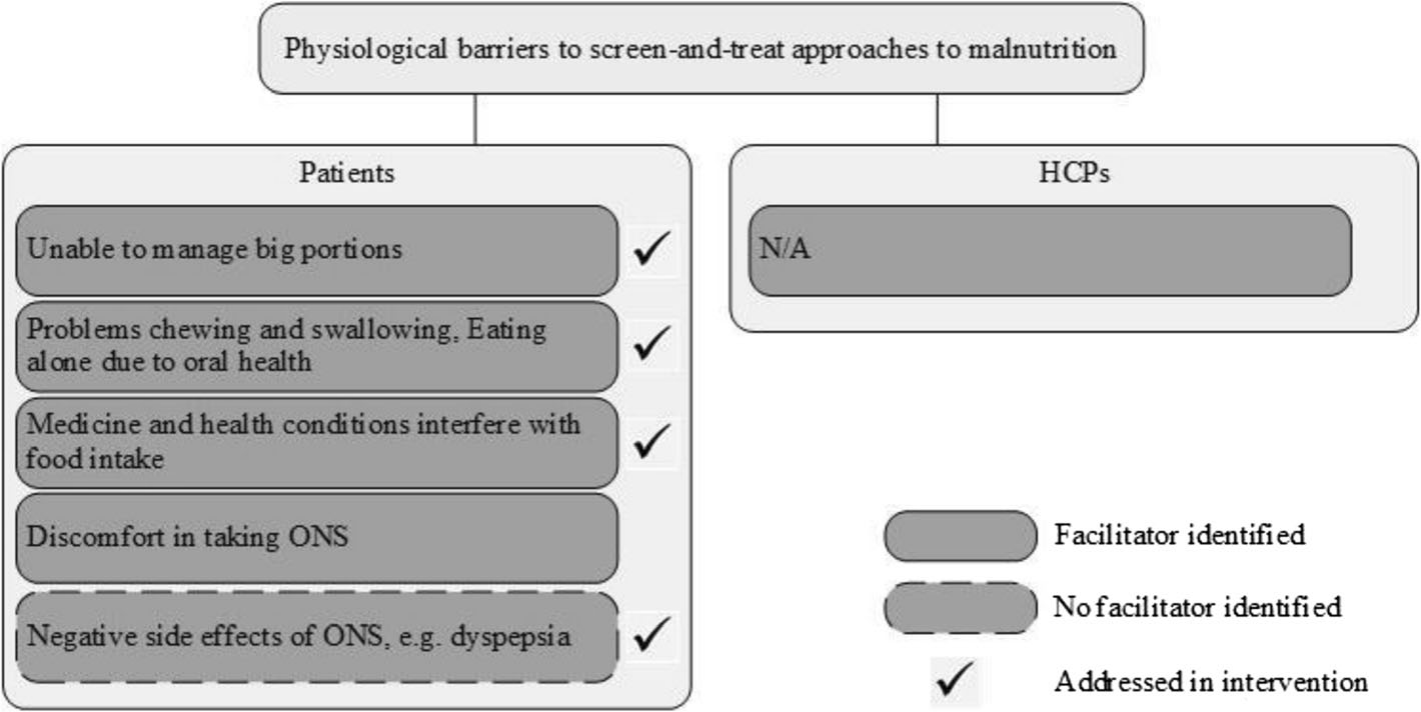
Physiological barriers to screen-and-treat approaches to malnutrition

### Critical appraisal

Studies were assessed using the Mixed-Methods Appraisal Tool (MMAT [35]). The MMAT differentiates studies based on how many quality criteria they meet: High quality studies meet at least 2 of 4 quality criteria, whereas low quality studies meet fewer than 2 criteria. LP and PH first trialled the MMAT on a small selection of papers. Overall, agreement was acceptable (76%), but some criteria were identified as ambiguous (criteria 1.3, 1.4, 2.3, 3.4 and 4.4). The raters agreed on a mutual understanding of these before each independently assessing all remaining studies.

## Results

Of the 21 included studies (Fig. 1), seven focused on HCPs and 14 on older people, who are referred to as ‘patients’, though some were not recruited or treated by HCPs; see Additional file 4: Tables S4-S5 for details of HCPs and patients. Around half of all studies (seven interventions and three qualitative) met MMAT criteria for high quality [35], however no low quality studies are excluded from the results presented below [36]. Results drawn from interventions deemed to be of higher or lower quality are summarised separately in Additional file 3: Tables S1-S3, to show which results are likely to be more reliable.

All extracted barriers and facilitators can be found in Additional file 3: Tables S1-S3 and all study characteristics can be found in Additional file 4: Tables S4-S5. Of note, the ten interventions targeting patients varied considerably in content. As detailed in Additional file 4: Table S4, seven [37–43] provided individual nutritional counselling from dietitians or nutritionists. In three of these [38–40], this was complemented with support from physicians, nurses, physiotherapists or occupational therapists, in a multi-disciplinary approach. In three other interventions [44–46], participants received nutrition: one intervention provided participants with ONS, one with food and one with snacks. The reported effectiveness of all interventions was varied and inconclusive, echoing previous reviews [24, 47]. For example, some of the nutritional counselling interventions showed some promising effects on body weight [37, 43] and physical functioning [37], whilst others did not [41, 42].

**Fig. 4:**
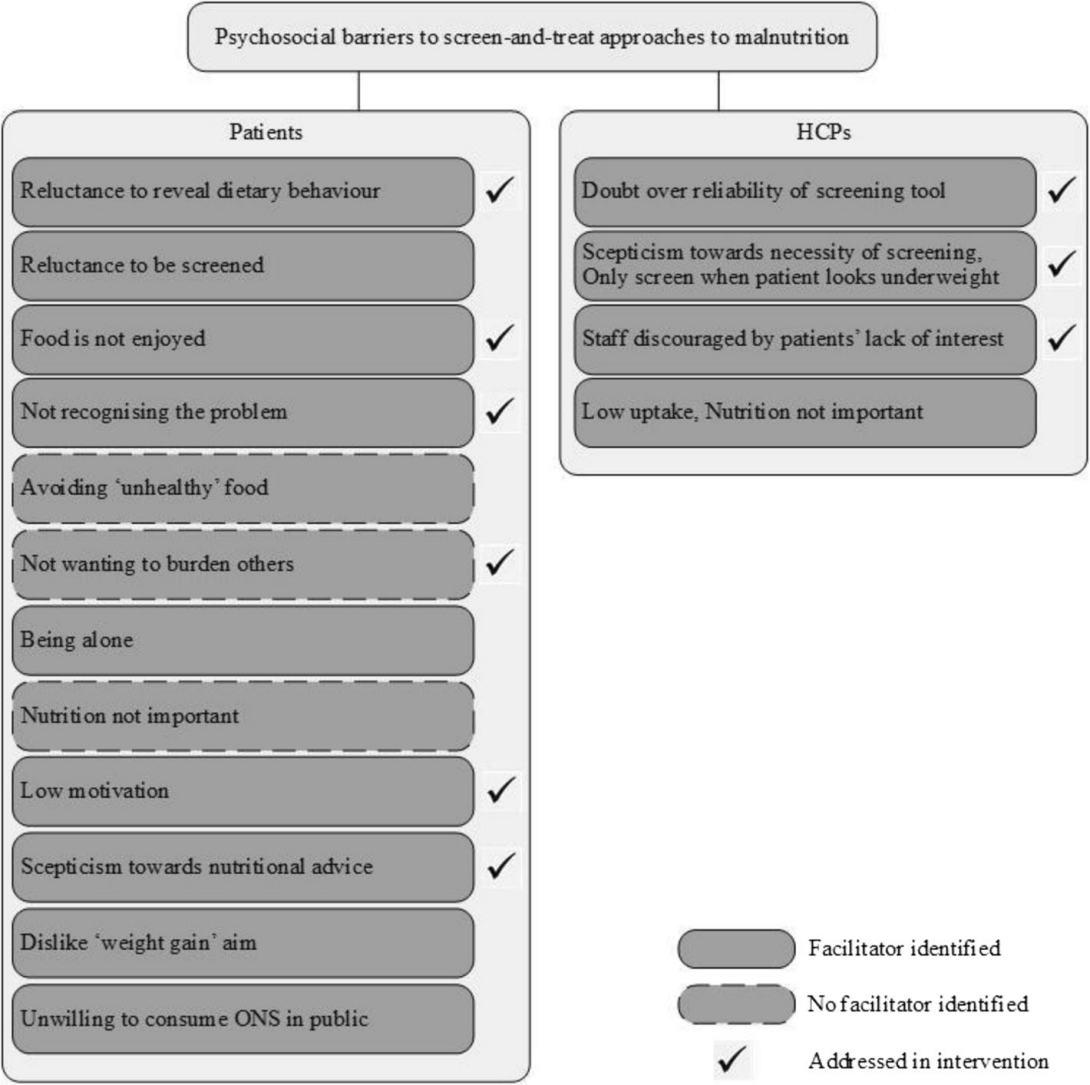
Psychosocial barriers to screen-and-treat approaches to malnutrition

Figures 2, 3 and 4 show whether interventions have incorporated the barriers and facilitators that emerged from qualitative studies. In the figures, these are separated by barriers and facilitators that patients and healthcare professionals may experience. In the following text, they are described together to emphasise areas where barriers and facilitators overlapped or differed.

### Barriers and facilitators to screening

Barriers to screening were common to both patients and HCPs: time taken to screen and reservations toward screening. Duration of screening was mostly addressed through shorter screening tools. The burden on HCPs’ time was additionally alleviated by patients filling in parts of the screener themselves, which seemed acceptable to patients and HCPs and mostly accurate (see Additional file 3: Table S1). Screening was not currently part of practice routine (see [28], but possible solutions included screening during routine appointments.

Patients were reluctant to describe their diet, for example because they were uncomfortable disclosing a poor diet [48], whereas HCPs had doubts over the need for and benefits of screening. Interventions educated HCPs on the purpose and importance of screening, but no intervention reported doing the same for patients. No intervention measured whether HCPs’ scepticism had been alleviated through training and only one intervention reported the number of patients who turned down screening (20% [46]).

### Barriers and facilitators to treating malnutrition

Patients perceived physiological and practical barriers to nutritional self-care (e.g. difficulties chewing, swallowing, shopping or preparing food). Multidisciplinary approaches addressed these by referring to the relevant specialist (e.g. dentist, physiotherapist or occupational therapist). Conversely, interventions that provided nutritional or dietitian counselling addressed physiological barriers, such as being unable to eat big portions, through self-help advice. Changes to eating behaviour, e.g. eating smaller portions or adding energy-rich food, was often central to these and appeared feasible and acceptable [37, 41–43].

Psychosocial barriers were the most frequent to not be addressed by interventions. More specifically, older adults may not consider nutrition as important, or fail to recognise the problem [48–51] because they perceive themselves as healthy, and consequently avoid ‘unhealthy’, energy-dense food [45, 50, 51] . No facilitators to these barriers emerged from the qualitative studies.

No intervention addressed the barrier of loneliness. Qualitative studies showed older adults may struggle with cooking [46, 49, 50] and eating alone [51]. A possible solution may be to offer ideas to help patients connect with others, but none of the interventions offered such self-help advice.

A further gap was how the intervention is presented to patients. Patients may be dissuaded from engaging if told that the aim is for them to gain weight, which may be perceived as aversive [52]. No intervention explicitly stated how the intervention was presented to patients.

Key barriers faced by HCPs were lack of time and low self-efficacy in malnutrition treatment pathways. Provision of written resources to alleviate burden placed on HCPs was a common feature of interventions and well-received by HCPs. Training to raise self-efficacy and build motivation for the importance of nutritional care was provided by only one high quality intervention [41]. No other solutions were identified in qualitative studies or tested in interventions.

### Barriers or facilitators to ONS uptake

Giving patients ONS is one treatment approach in the reviewed studies. No interventions recorded (by measuring compliance) whether patients were persuaded to consume ONS. Of note, in the intervention where ONS uptake resulted in improved weight and physical function [45], participants received clear instructions on how to take ONS, which no others reported. A notable psychosocial barrier was that patients may be reluctant to consume it publicly due to unwanted attention. A possible facilitator mentioned was to normalise consumption [53], by treating ONS as food not medicine, but interventions did not address this.

HCPs had reservations about prescribing ONS. These reservations were only addressed in one intervention [54] (deemed low quality), despite ONS frequently being a component of interventions. It is not yet clear what an effective training programme for HCPs needs to incorporate, but simple solutions have been proposed such as explaining that appropriate prescribing can save money (Fig. 2).

## Discussion

### Summary

This synthesis identified, from recent literature, barriers and facilitators to screening and treating malnutrition in community-dwelling older adults in primary care, and demonstrated whether and how interventions have incorporated these. The studies document numerous physiological, practical and psychosocial barriers to patients’ and HCPs’ engagement with screening and treating malnutrition, but our novel approach to mapping these onto intervention features revealed the following gaps: interventions did not address patients’ scepticism about malnutrition screening, endeavour to increase readiness to be screened (e.g. through education) or measure reactions to screening. We currently have little data on how older adults perceive screening or why they are reluctant to be screened [48, 49]. Notably, findings relating to patients’ barriers to screening emerged largely from HCPs’ experiences [48, 49, 55]. Moreover, we noted some conflicting findings, such as that some patients are willing to be screened when the purpose of screening is explained to them [55], whilst others seem to prefer not to know [55]. Similarly, some patients in a qualitative study were surprised or offended to be told they were ‘at risk’ after screening, while others were unconcerned [56]. Such differences may be due to preferences of individual patients, their experience of the patient-practitioner relationship or the way that risk information is conveyed. Further studies exploring older patients’ experience of being screened in primary care are needed to promote and support their self-management and identify effective ways to convince patients of the value of screening.

Practical and physiological barriers and facilitators to nutritional self-care were incorporated in the interventions reviewed, and steps taken to overcome these barriers are in line with those suggested by care pathways for the management of disease-related malnutrition [17]. However, a prominent gap was in considering psychosocial barriers, which may link to psychosocial causes of malnutrition [2]. These included loneliness, and patients perceiving themselves as healthy and avoiding ‘unhealthy’ food, highlighting the potential benefit of screening regardless of whether patients report any health issues. A recent randomised controlled intervention study identified additional beliefs that interfered with patients’ adoption of self-care components, including not believing that the recommended action would solve the problem [57].

A psychosocial barrier to engaging in nutritional interventions may be how an intervention is presented to patients (e.g. whether its aim is ‘weight gain’). Interventions did not explicitly report how they were presented to patients, but it could be a factor that may promote or hinder engagement. Van der Pols-Vijlbrief and colleagues [57] also suggest that easy-to-execute actions such as tips promoting three or more snacks a day and increased physical activity may be adopted more readily.

Previous research shows ONS to be effective in hospital patients in terms of weight gain [22], reduced complications and mortality, and may be effective in community settings, including care homes, sheltered housing or among free-living older adults, particularly when ONS is initiated during a hospital stay [58]. However, good quality prospective studies are needed to establish whether ONS is beneficial when initiated in primary care [59]. Future studies are needed to test whether ONS can make a difference to the nutritional status of free-living older adults who are at risk, but who have not yet had an acute episode that triggers malnutrition screening. However, this is unlikely to address the underlying issue of patients not recognising the problem, for example where malnourishment is related to social factors [2]. In order to test the effectiveness of ONS in the community, HCPs need to be convinced of the need to test the potential value of ONS and to prescribe according to protocol. Our synthesis therefore emphasises that interventions need to address engagement of HCPs and patients with the idea of prescribing or consuming ONS to treat malnutrition where necessary, otherwise tests of the effectiveness of ONS may not be valid. HCPs’ reservations need to be countered, and patients need to be given practical and psychological support to enhance consumption. For example, ONS may be uncomfortable to consume, though no intervention in this synthesis considered this, but which could be addressed through practical advice (e.g. drinking through a straw). Results showed that interventions providing patients with ONS rarely reported incorporating such education or support. It seems theoretically possible that informed education on the benefits of ONS for HCPs could help, but for this to be effective, further research is needed in order to explore and address the underlying reasons for their reservations.

### Strengths and limitations

This synthesis highlights how considering qualitative data alongside quantitative data may help explain quantitative findings and can lead to different conclusions than considering each in isolation [60]. First, those studies with mixed-methods approaches provided the richest findings, e.g. documenting patients’ reasons for discontinuing an intervention [44], which can help improve future interventions [61]. Second, the mixed-methods approach of this synthesis allowed for greater scope and insights into whether interventions can address older, community-dwelling adults’ barriers to nutritional self-care.

Interventions tended to be complex (thus making it difficult to isolate the active ingredient), to involve small, diverse samples, and to vary substantially (e.g. in their duration and geographical location). Some baseline variables, such as HCPs’ existing levels of nutrition knowledge, were unknown. This heterogeneity precluded a meta-analytic approach to quantifying effects and made direct comparisons across studies difficult. However, as the number of interventions being trialled is steadily growing, the available evidence may soon be rich enough to conduct such meta-analyses.

We included only studies published since the Cochrane review on dietary counselling and ONS [33], yet barriers and facilitators to screen and treat may have been identified in studies published prior to 2012. However, only four studies identified by Baldwin et al. [33] focused on community-dwelling older adults, and we considered that practice is likely to have changed since these publications from 1985, 1995, 2003 and 2008.

A further limitation was the quality of included studies. Around half the studies were judged to be of low quality and conclusions drawn from these must be treated with caution. This concurs with other reviews on malnutrition interventions [14, 15, 45, 62, 33]. It is noteworthy, however, that low scores on the MMAT were often due to reviewers having to assign the category ‘Can’t Tell’ (in 18% of classifications). The MMAT is a relatively new tool designed to assess the quality of a number of study types, and the number of ‘can’t tell’ classifications we made may indicate that improvements are needed. Thus, studies may have been well designed, but insufficient reporting and / or limitations of the MMAT reduced our ability to judge study quality, highlighting the importance of adhering to accepted reporting standards (e.g. [63]). Insufficient reporting further limited our ability to judge whether some interventions incorporated named facilitators, such as providing evidence on the effectiveness of screening in HCPs’ training.

### Comparison with existing literature

Although the synthesis makes an important contribution by identifying key barriers, possible solutions and areas where future interventions must be targeted, it is not yet possible to identify the key ingredients of an effective intervention. We calculated effect sizes where possible (Additional file 4: Table S4), but only a few studies reported the relevant statistics, limiting our ability to compare and judge effectiveness. This echoes previous reviews on malnutrition interventions targeting older, community-dwelling adults [24, 47] and the most recent clinical guidelines in the UK [11].

The findings regarding HCPs’ barriers and facilitators to screening show coherence with the results of a previous review [28]. The results further strengthen the argument that screening alone is insufficient [26, 64] and must be accompanied with appropriate nutrition care pathways.

### Implications for research and practice

When intervention targets (e.g. ONS consumption) are not met, the effectiveness of an intervention should be questioned [47, 65]. Two points follow on from this: first, this could explain some of the inconsistent effects observed in this synthesis, as compliance varied overall (and was not reported for ONS). Second, participation in screening should be considered a crucial aspect of intervention fidelity. As this synthesis demonstrates, screening harbours its own set of barriers for both HCPs and patients, and thus it is informative to know how both reacted to screening. Studies should report the number of patients who refused screening (which only one study in this synthesis did [46]). It would be informative to explore patients’ perceptions of screening and speak to those who refuse screening [66, 67].

## Conclusion

In this synthesis we have identified multiple barriers to implementing screen and treat policies in primary/community care for both HCPs and patients. We have also identified possible facilitators to address these barriers, both from studies exploring HCPs’ and patients’ perspectives and from previously tested interventions. We have also identified barriers that were not addressed within the reviewed interventions, but which could be addressed with well-designed intervention features (e.g. addressing misconceptions about ‘unhealthy’ food for older adults through education and overcoming HCP scepticism for screening). Future interventions need to be developed with the complex barriers of both HCPs and patients in mind. Research is now needed to establish whether interventions designed to address the identified barriers to screening and treatment of malnutrition are effective.

## Additional files

Additional file 1: Search strategy. (DOCX 13 kb)

Additional file 2: Inclusion and exclusion criteria. (DOCX 14 kb)

Additional file 3: **Table S1.** Synthesis matrix for screening for malnutrition. **Table S2.** Synthesis matrix for treating malnutrition. **Table S3.** Synthesis matrix for prescribing or taking ONS. (DOCX 24 kb)

Additional file 4: **Table S4.** Characteristics of interventions. **Table S5.** Characteristics of qualitative studies. (DOCX 31 kb)

## Data Availability

All data generated or analysed during this study are included in this published article and its supplementary information files.

## Abbreviations

HCPs: Healthcare professionals
MMAT: Mixed Methods Appraisal Tool
MUST: Malnutrition Universal Screening Tool
ONS: Oral nutritional supplements
RCT: Randomised controlled trial
