# Supplementary material for: Barriers and facilitators to screening and treating malnutrition in older adults living in the community: a mixed-methods synthesis
Source: BMC Fam Pract. 2019 Jul 15;20:100. doi: 10.1186/s12875-019-0983-y (PMC6631945; doi:10.1186/s12875-019-0983-y)
Supplement: Supplementary file 1 — Search strategy. (DOCX 13 kb) [file 12875_2019_983_MOESM1_ESM.docx]

**Search strategy**

Search carried out in Medline, then adapted for EMBASE, PsychINFO, CINAHL, Cochrane Central Register of Controlled Trials, Cochrane Database of Systematic Reviews and DARE

| **Database and date** | **Search terms** |
| --- | --- |
| OVID Medline 1996 to June 2016 | 1. Community.ti,ab.  2. Primary Health Care/  3. exp Community Health Services/  4. exp General Practice/  5. primary care.ti,ab.  6. 1 or 2 or 3 or 4 or 5  7. home.ti,ab.  8. free living.ti,ab.  9. 6 or 7 or 8  10. (screen* adj tool*).ti,ab,hw.  11. (screen adj2 treat*).ti,ab,hw.  12. MST.ti,ab.  13. (nutrition* adj assessment*).ti,ab,hw.  14. (nutrition* adj screen*).ti,ab,hw.  15. (screen* adj checklist).ti,ab,hw.  16. (nutrition* adj questionnaire).ti,ab,hw.  17. ONS.ti,ab.  18. (nutrition* adj supplement*).ti,ab,hw.  19. (assessment adj tool*).ti,ab,hw.  20. protein*.ti,ab.  21. calorie*.ti,ab.  22. (energy adj intake).ti,ab,hw.  23. (diet* adj2 supplement*).ti,ab,hw.  24. (oral adj2 nutrition*).ti,ab,hw.  25. (oral adj2 supplement*).ti,ab,hw.  26. sip feed.ti,ab.  27. (nutrition* adj2 intervention*).ti,ab,hw.  28. (diet* adj2 intervention).ti,ab,hw.  29. diet therapy.ti,ab,hw.  30. Diet Therapy/  31. Nutrition Therapy/  32. 10 or 11 or 12 or 13 or 14 or 15 or 16 or 17 or 18 or 19 or 20 or 21 or 22 or 23 or 24 or 25 or 26 or 27 or 28 or 29 or 30 or 31  33. (screen* or treat*).ti,ab.  34. 32 or 33  35. 9 and 34  36. (malnutrition or malnourish* or nutrition* or under-nutrition or dehyrat* or hydrat*).ti,ab,hw.  37. Malnutrition/  38. Protein-Energy Malnutrition/  39. 36 or 37 or 38  40. 35 and 39  41. limit 40 to English language  42. limit 41 to humans  43. limit 42 to yr=”2012 –Current” |
