# Supplementary material for: Barriers and facilitators to screening and treating malnutrition in older adults living in the community: a mixed-methods synthesis
Source: BMC Fam Pract. 2019 Jul 15;20:100. doi: 10.1186/s12875-019-0983-y (PMC6631945; doi:10.1186/s12875-019-0983-y)
Supplement: Supplementary file 2 — Inclusion and exclusion criteria. (DOCX 14 kb) [file 12875_2019_983_MOESM2_ESM.docx]

Additional file 2 Inclusion and Exclusion criteria

***Inclusion criteria***

| **Population** | *Older adults:*  Adults aged >= 65 yrs and living in the community  (mean age of 75 if a range including those below 65) | *Healthcare professionals:*  Healthcare professionals who would care for older, community-dwelling adults |
| --- | --- | --- |
| **Setting** | Primary care AND/OR Community | |
| **Intervention** | Screening AND/OR treatment for risk of malnutrition  (Only validated screening tools for community settings - outpatients, at home, in general practice, other primary care) | |
| **Comparator** | Any comparator or none – for comparison studies (e.g. RCTs) we will evaluate interventions and intervention components with respect to the comparison chosen by the researchers | |
| **Outcomes**  (validated measures) | *Older adults:*  Quality of life, including:   - Nutritional status (e.g. diet, eating habits, malnutrition tool) - Physical functioning (e.g. grip strength, chair stand, mobility) - Mood - Satisfaction (e.g. ease of use) - Hospital admission | *Healthcare professionals:*   - Compliance with screen and / or treat - Satisfaction (e.g. ease of use) |
| **Study design** | - RCTs / cross-sectional / cohort / observational / feasibility studies /pilot studies / qualitative studies (experiences / views of patients, carers and health professionals) / implementation of Malnutrition Screen and Treat policies | |

***Exclusion criteria***

- Nursing homes, residential care, care homes, hospital inpatients
- Comparative studies of nutrition screening tools, validation studies, reviews
- Terminal disease, ongoing primary treatment for cancer, established dementia, diabetes
- Parenteral nutrition
- Languages other than English
