# Supplementary material for: Barriers and facilitators to screening and treating malnutrition in older adults living in the community: a mixed-methods synthesis
Source: BMC Fam Pract. 2019 Jul 15;20:100. doi: 10.1186/s12875-019-0983-y (PMC6631945; doi:10.1186/s12875-019-0983-y)
Supplement: Supplementary file 3 — Table S1. Synthesis matrix for screening for malnutrition. Table S2. Synthesis matrix for treating malnutrition. Table S3. Synthesis matrix for prescribing or taking ONS. (DOCX 24 kb) [file 12875_2019_983_MOESM3_ESM.docx]

Additional file 3: Tables S1-S3

| *Table S1.* Synthesis matrix for screening for malnutrition | | | | |
| --- | --- | --- | --- | --- |
| Barriers and facilitators | |  | Interventions which address barriers or facilitators | |
| Barriers | Facilitators |  | Soundly evaluated interventions (met 50% or more of MMAT criteria) | Other evaluated interventions (met less than 50% of MMAT criteria) |
| **Barriers or facilitators to screening for malnutrition: patients** | | | | |
| Time taken to complete screening off-putting^45,54^ | Screening with six questions is acceptable^54^ |  | Use Short Nutritional Assessment Questionnaire, which has 4 items^40^  Use Nutritional Risk Screening 2002, which has 5 items^36^  Use Malnutrition Screening Tool, which has 3 items^68^ | Use Malnutrition Universal Screening Tool, which has 4 items^53^  Use Mini-Nutritional Assessment-Short Form, which has 6 items^54^ |
| Reluctance to reveal dietary behaviour^47^ | Patients unaware of being screened if integrated into other assessment^54^ |  | Present screener together with gait speed test^44^  Present screener together with questions unrelated to nutrition^41^ | Present screener together with questions unrelated to nutrition^37^ |
| Reluctance to be screened^47,48^ | Patients willing if purpose of screening is explained^54^ |  | Not addressed | Not addressed |
| **Barriers or facilitators to screening for malnutrition: HCPs** | | | | |
| Lack of resources (staff, time, space, money)^47,69,70^ | Patients self-complete where possible^36^  Clearly defined responsibilities^47,53,69,70^  Screen patients in the GP waiting room (where they spend on average 21 minutes)^47^ |  | Not addressed | Patients self-completed MNA-SF, with 12% being misclassified. Patients tended to score themselves as higher risk than did practitioners. Errors occurred with weight loss and BMI calculation^54^.  Offer electronic versions of resources to print^54^  Keep resources concise^54^ |
| Staff make mistakes with screening tool^47,68^  Lack of familiarity^47^  Lack of training^47,69,70^ | Provide training in how to screen^47,54,69,70^ |  | Education session^68^ | Group training^53,54^  Reinforce knowledge through questionnaires^53,54^  Consistency in training^53^ |
| No time or money for training^70^ | None identified |  | Use an online training tool^68^ | Local training session^54^ |
| Screening tool difficult to use^69^ | Familiarity increases ease of use^69^ |  | Monitor accuracy in screener completion and provide feedback^68^ | Not addressed |
| Measuring height or weight impractical^36,40,69^ | None identified |  | Not addressed | Train in alternative measurements^53^ |
| Doubt over reliability of screening tool^47,69^ | Use validated screening tool^48,54,68,69^ |  | Malnutrition Screening Tool^68^ | Malnutrition Universal Screening Tool^53^  Mini-Nutritional Assessment – Short Form^54^ |
| Scepticism towards necessity of screening^47,48,69^  Only screen when patient looks underweight^68,69^ | Inform about benefits of screening^68,70^   - Importance of managing weight^70^ - Screener is a record of care^69^   Screening identifies malnutrition even if not obvious^54,71^  Screening tools can be an objective and non-threatening way to assess nutritional risk^54^ |  | Provide information on malnutrition^68^  Screen all patients on admission^68^ | Educate about causes^53^  Provide underlying theory^53^ |
| Low uptake of training^68^ | Make training accreditable^53^ |  | Follow-up with staff who missed training^68^  “Quality Improvement Activity”^68^ | Visual training materials^53,54^  Interactive learning^53^ |
| Low uptake of screening^69^ | None identified |  | Not addressed | Provide ready-to-use screeners^54^  Provide screening tool^53^  Encourage screening^53^  Offer education programme to all staff in practice^53^ |
| Lack of awareness^47,70^  Lack of confidence to do screening^69,70^ | Raise awareness^70^  Training increases knowledge and confidence^69^ |  | Provide printed information^68^ | Provide information booklet for practitioner^54^  Can report difficulties^53^  Reinforce knowledge on screening and treating malnutrition through questionnaires^53,54^ |
| Screening not integrated into practice^40,47,68,69^ | Make screening part of routine practice^39,47,48,668,69^   - Screen during routine appointments^47,54,68^   - Integrate screener into software^54^ |  | Screen on admission to care^68^  Screener integrated into documentation^68^ | Screen during routine appointment^54^ |
| Staff discouraged by patients’ lack of interest^47^ | Use case studies to share experience^69^ |  | Raise awareness of patients’ reluctance^68^ | Provide case studies^53,54^ |
| Lack of care pathways to guide action after screening^48,69^ | Effective communication of screening results^48,69^ |  | Care pathway with actions based on screening result^68^  Referral pathways (e.g. to dietitian, to dentist, to physiotherapist)^37-39^ | Provide reminder of care pathways^54^  Refer to dietitian^53,54^ |

Notes: BMI = Body Mass Index; MMAT = Mixed Methods Appraisal Tool; MNA-SF = Mini Nutritional Assessment-Short form

| *Table S2.* Synthesis matrix for treating malnutrition | | | | |
| --- | --- | --- | --- | --- |
| Barriers and facilitators | |  | Interventions which address barriers or facilitators | |
| Barriers | Facilitators |  | Soundly evaluated interventions | Other evaluated interventions |
| **Barriers or facilitators to nutritional self-care** | | | | |
| Difficulties shopping^43,48,50,49^  Cost of eating^43,48,49^ | Use walking aids^50^  Have food delivered^50^  Keep physically active^50,49^ |  | Refer to physiotherapy^39^ | Refer to physiotherapy^37,38^  Provide ingredients for cooking^45^  Provide snacks^43^  Discuss social assistance^37^ |
| Difficulties preparing food^45,48,49^ | Eat meals out^50^  Have simple meals^50^  Cook in bulk and freeze portions^49^  Ability to cook^50^ |  | Encourage snacking^39^ | Encourage snacking^43^  Advice on cooking techniques^37^  Home modifications to help with visual or auditory impairments^37^  Provide recipes with videos^45^  Discuss social assistance^37^ |
| Food is not enjoyed^45,50,49^ | Putting effort into making food attractive and enjoyable^50,49^  Enjoy food from childhood^49^ |  | Identify food preferences^41^  Provide recommendations for taste changes^40^  Meal suggestions (inspirations)^40^  Tailored meal plans^39^ | Adapt to taste of participant^43^  Focus on preparing attractive meals^50^  Food diary analysis^37^  Recommend exercise before eating^37^ |
| Unable to manage big portions^48,71^ | Eat little, but regularly^48,49,50^ |  | Increase frequency of meals^36,39^  Food fortification^36,39-42^ |  |
| Problems chewing and swallowing^39,50^  Eating alone due to oral health^50^ | Address physical barriers^42^ |  | Not addressed | Advice on adaptation of textures^37^  Refer to dentist^37^  Refer to occupational therapist^38^ |
| Medicine and health conditions interfere with food intake^50,49^ | Management of side effects of medication^49^  Physical activity prevents obstipation^49^ |  | Provide recommendations for dealing with nausea^40^ | Review chronic prescriptions^37^  Suggest non-pharmacological options to manage sleep, pain, constipation^37^  Recommend exercise before eating^37^ |
| Not recognising the problem^47,51,50,49^ | Proactive and open to nutritional support^47,50,49^ |  | Education by dietitian^36^  Give ONS^40,44^ | Education by dietitian^38^  Give ONS^37,51^  Monitor progress and adherence to raise awareness^36,37,39,41^ |
| Avoiding ‘unhealthy’ food^44,50,49^ | None identified |  | Not addressed | Not addressed |
| Not wanting to burden others^41^ | None identified |  | Involve caregivers^41^  Involve family members^39,42^ | Information on nutrition for carers^37,38^ |
| Life changes^37,61^ | None identified |  | Assist with problem solving (no further details given)^38^ | Discuss depression^34^ |
| Lack of routine^50^ | Make cooking part of daily routine^49^  Plan ahead^49^ |  | Focus on patient’s habits and patterns^41^  Identify self-efficacy to make changes^41^  Make plans and set goals^40,41^ | Not addressed |
| Being alone^50^ | Eat with others^49^  Eating alone can be peaceful^49^ |  | Not addressed | Not addressed |
| Nutrition not important^51,48^ | Remind of importance of nutrition^50,49^  Remind of link between eating and wellbeing^50^ |  | Not addressed | Not addressed |
|  | Knowledge of importance of fluid intake^49^ |  | Not addressed | Monitor water intake^45^ |
| **Barriers or facilitators to delivering nutritional self-care interventions: HCPs** | | | | |
| Lack of time^53^ | Hand out resources preferred^54^ |  | Printed info for patients^36,39-42^ | Printed info for patients^37^ |
| Low uptake^36,70^  Nutrition not important^47^ | Clarify role of HCP in nutrition care^70^ |  | Not addressed | Not addressed |
| No confidence in personal effectiveness^54,70^  No consensus on best approach^47,70^ | Provide training in care pathways^37,69^ |  | Training in treating malnutrition in older adults^40^ | Training in risk of malnutrition in older adults^50^ |
| **Barriers or facilitators to engaging in nutritional self-care interventions: Patients** | | | | |
| Low motivation^40,70^ | Minimise effort for patient^41^ |  | Home visits by dietitians aimed at maximising motivation^36^  Discuss motivation^41^  Avoid overwhelming with information^41^  Praise success^41^  Amend plans and goals based on progress to enhance follow-through^39,41^  Telephone follow-up^36,39,41,42^ | Provide ingredients for cooking^45^  Provide snacks^43^  Allow feedback on usefulness^45^ |
| Scepticism towards nutritional advice^49^ | Understandable, written content^50^ |  | Intervention administered by dietitian^39,41^ | Explain function of nutrients^45^  Check understanding of instructions^45^ |
| Not enough food suggestions^45^ | Provide many simple recipes^45^ |  | Not addressed | Suggest variations of recipes^45^  Check liking of food suggestions^45^  Different snack every day^43^ |
| Dislike ‘weight gain’ aim^41^ | Avoid marketing study as 'weight gain'^41^ |  | Not addressed | Not addressed |
|  | Holistic approach to malnutrition^40,70^ |  | Consultation with both dietitian and GP^36^ | Not addressed |
|  |  |  |  |  |

| *Table S3.* Synthesis matrix for prescribing or taking ONS | | | | |
| --- | --- | --- | --- | --- |
| Barriers and facilitators | |  | Interventions which address barriers or facilitators | |
| Barriers | Facilitators |  | Soundly evaluated interventions | Other evaluated interventions |
| **Barriers or facilitators to ONS uptake: Patients** | | | | |
| Little awareness of purpose^51,71^ | Inform about purpose of ONS^51,71^  Discuss ONS initiation with patients^51^  Trust in prescriber^71^ |  | ONS recommended by dietitian^36^ | Explain that ONS do not replace food^44^ |
| Discomfort in taking ONS^51^ | ONS convenient to consume through straw^71^ |  | Not addressed | Not addressed |
| Negative side effects, e.g. dyspepsia^44^ | None identified |  | Dietitian home visit after one week, and regular telephone contact^36^ | Regular follow up, e.g. three-monthly^37^  Weekly discussion of patient^38^  Monitor patient daily^51^  Offer easy opportunity to contact HCP^51^ |
| Unwilling to consume ONS in public^71^ | Normalise ONS as food, not medicine ^51,71^ |  | Not addressed | Not addressed |
| Lack of choice of ONS products^71^ | Offer different products and flavours^44,53^ |  | Not addressed | Offer different flavours^38,51^ |
|  | Compliance influenced by person factors (e.g. reason for taking ONS)^71^ |  |  |  |
| **Barriers or facilitators to prescribing ONS: HCPs** | | | | |
| Cost^48^ | More appropriate prescribing can save money^53^ |  | Not addressed | Prescribe ONS after full dietetic assessment^53^ |
| Suboptimal referring and monitoring^53^ | Provide ONS education^53^ |  | Not addressed | Educate on ONS^53^  Monitor ONS compliance^44,45,51^  Provide telecare monitor to monitor adherence^51^ |
| Hard to wean patients off ONS^48^ | None identified |  | Not addressed | Not addressed |

Note: ONS = Oral Nutritional Supplements
