# Supplementary material for: Barriers and facilitators to screening and treating malnutrition in older adults living in the community: a mixed-methods synthesis
Source: BMC Fam Pract. 2019 Jul 15;20:100. doi: 10.1186/s12875-019-0983-y (PMC6631945; doi:10.1186/s12875-019-0983-y)
Supplement: Supplementary file 4 — Table S4. Characteristics of interventions. Table S5. Characteristics of qualitative studies. (DOCX 31 kb) [file 12875_2019_983_MOESM4_ESM.docx]

Additional file 4: Tables S4 and S5

| *Table S4.* Characteristics of interventions. | | | | | | | | | |
| --- | --- | --- | --- | --- | --- | --- | --- | --- | --- |
| Reference, country, design | Population | Screening tool | Sample size | Intervention (and control group if applicable) | ONS used (if any) | Outcomes, effect size where possible to calculate | Follow-up | MMAT | Author conclusions regarding effectiveness |
| Craven et al. (2014)^68^  Australia  Pre-post | Healthcare professionals  Nurses and staff members | MST | 22 | 1. Education session, which provided an overview of the issue of malnutrition in the community, instructions on how to complete the MST and on referral pathways | None | - Use of MST - Initiation of action plans - Screening new clients on admission - Accurately completing MST | 4 months | 3-4* | “Following the implementation phase there was significant improvement in compliance across all criteria.” |
| Hamirudin et al. (2014)^54^  Australia  Mixed: Feasibility and interviews | Healthcare professionals  General practitioners, general practice registrars and practice nurses | MNA-SF | 22 | 1. In-practice, dietitian-led group workshops on using the MNA-SF | None | - Feasibility of MNA-SF - usefulness of resource kit - nutrition knowledge and ability to identify malnutrition in patients | 3 months | 0-2* | “Knowledge scores of staff significantly improved from baseline” |
| Kennelly et al. (2011)^53^  Ireland  Pre-post | Healthcare professionals  GPs, practice nurses, community nurses and nursing home staff (only data from community used) | MUST | 96 | 1. Individually tailored, dietitian-led nutrition education, provision of a resource folder | None | - Use of structured screening - Referral to dietetics - Nutritional care actions - Selecting those suitable for ONS | 1 year | 0-2* | “One year later, screening for malnutrition risk was better, dietary advice was provided more often, referral to the community dietetics service improved and ONS were prescribed for a greater proportion of patients at ‘high risk’ of malnutrition than before”. |
| Badia et al (2015)^37^ Spain  RCT | **Patients**  Living alone, with comorbidities (mostly hypertension) | MNA | 328 | **Multidisciplinary approach**  1. Treatment plan targeting 9 malnutrition risk factors (psychotropic and cardiovascular drug use, auditory and vision acuity, balance and gait disorders, nutrition, disability, cognitive impairment, social risk and home safety). FU visit/phone call every 3 months. Nutritional and rehabilitation assessment during second year. High risk received 3 one-hour individual meetings with a dietitian who developed a plan for individualized nutrition (incl. diary analysis, advice on dietary adaptation to address the most common nutritional problems, recommendations regarding basic cooking techniques, adaptation of textures, or ONS if in need of extra calories). 2. Usual care | Not specified: Nutritional supplement | - Changes in MNA - Changes in diagnosis of anaemia - Functional status - Analytical parameters - Hospital admissions/ emergency hospital visit - Quality of Life (EuroQol-5D) | 12 months 24 months | 0-2* | “The main result of the present study is that a multifactorial assessment with individual intervention in the oldest old people living in a community-dwelling has not provided evidence of effectiveness in nutritional status.” |
| Beck et al. (2013)^36^  Denmark  RCT | **Patients**  Recently discharged from hospital | Nutritional Risk Screening 2002 (NRS2002) | 152 | **Nutritional advice**  1. 3 x post-hospital FU with GPs (inc nutrition check) 2. 3 x post-hospital FU with GPs (inc nutrition) plus 3 x nutrition counselling with dietitian (development of a nutritional plan which could include prescription of ONS if necessary), plus optional phone FU | Not specified: “Commercial oral nutritional supplements” | - Risk of readmission - Functional status (grip strength, *d* = 0.03, chair stand, *d* = 0.00, mobility, *d* = 0.26, disability, tiredness in daily activities, *d* = -0.06, rehab capacity) - Nutritional status (weight, *d* = 0.46, BMI, energy, *d* = 0.78 and protein intake, *d* = 0.55) - Need of social services (home care, home nursing, MOW) - Mortality | 12 weeks | 3-4* | The intervention “had a positive effect on functional status (i.e. mobility) and nutritional status (i.e. weight, energy and protein intake) but no effect on risk of re-admissions or mortality”. |
| Beck et al. (2016)^38^  Denmark  RCT | Patients  A mix of adults residing in nursing homes and receiving home-care. | Eating Validation Scheme | 246 (106 from home-care) | **Multidisciplinary approach**  1. Nutrition coordinator training plus multidisciplinary nutrition support from physio, occupational therapist, dentist, nurse, dietitian 2. Nutrition coordinator training plus usual care | Not specified: “Oral training supplement” | - Quality of Life (EQ5D 3L), *d* = 0.76 - 30-second chair stand, *d* = 0.67 - Weight, *d* = 0.15 - Hand grip strength, *d* = -0.13 - Oral care, *d* = -0.66 - Fall incidents - Hospital admission - Rehabilitation - Move to nursing home - Mortality | 11 weeks | 0-2* | “The present study suggests a positive effect of multidisciplinary nutritional support for undernutrition in elderly adults in nursing homes and home-care, identified with 2 points according to EVS on quality of life, muscle strength, and oral care.” NB: results hold separately for both nursing home residents and those receiving home care |
| Charlton et al. (2013)^43^  Australia  Mixed: Feasibility and interviews | Patients  Recipients of MOW, about half living alone, malnourished or at risk of malnutrition | MNA | 12 | **Provision of nutrition**  1. For a period of four weeks each participant received a snack for Monday through Friday, in addition to their standard MOW delivery. | None | - MNA - Height and weight, BMI - Protein and energy intake (through 24 hour food diary) - Semi-structured interview about experience of intervention | 4 weeks | 0-2* | “Provision of one nutritious, tasty snack per day to the study participants resulted in increases in weight and BMI over four weeks.” |
| Chung & Chung (2014)^45^  Hong Kong  Pre-post | Patients  Integrative health centre users | MNA | 60 | **Provision of nutrition**  1. 1 day food pack per week over 3 weeks plus group weekly cooking seminar 2. 3 x 1 day food pack per week over 3 weeks plus group weekly cooking seminar | None | - MNA - Patient satisfaction - Compliance - Patients' perceptions of ease of use - Willingness to continue cooking | 6 months | 0-2* | “The compliance rate of Group 2 was higher than that of Group 1. More than 60% of the participants intended to continue cooking and a third of the participants expressed satisfaction with the program. The MNA scores had improved 6 months later (combined data from both groups)” |
| Kim & Lee (2013)^44^  Republic of Korea  RCT | Patients  Low socioeconomic status, frail, half living alone | MNA | 87 | **Provision of nutrition**  1. 400 mL ONS per day for 12 weeks (400kcal, 25g protein, 9.4g essential amino acids) 2. No ONS | Greenbia HP, Dr. Jung’s Food Co., Ltd., Korea | - Timed get up and go, *d* = -0.25 - One leg stance, *d* = -0.32 - Grip strength, *d* = -0.26 - 3 x 24 hr diet recall - Nutrient adequacy ratio, *d* = 1.44 - Energy intake, *d* = 0.77, protein intake, *d* = 1.32, essential amino acids, *d* = 1.58 - Body weight *d* = 0.37, mid-arm circumference, *d* = 0.56 | 12 weeks | 3-4* | “This study demonstrates that protein-energy supplementation applied to frail older adults with low SES increases dietary intake and shows evidence of reducing the progression of functional decline.” |
| Kraft et al. (2012)^51^  Germany  Feasibility | Patients  Malnourished patients with a need for caloric ONS | 1. Nutritional Risk Score 2. The Geriatric Nutritional Risk Index | 26 | **Provision of nutrition**  1. ONS plus intensive self-monitoring via telemed after training. Tele-monitor alerted tele-medical centre to adverse events (e.g. patients not consuming ONS, not feeling well) 2. Usual care | Not specified: “Oral nutritional supplements” | - Nutrition Risk Screening score - BMI, *d* = 0.05 - Weight loss, *d* = -0.21 - Phase angle (Bioelectrical impedence Analysis) - Geriatric Nutritional Risk Index | 6 months | 0-2* | “Follow up data of body weight and BMI showed no relevant differences between both groups.” |
| Locher et al. (2013)^41^  USA  Feasibility  B-NICE trial | Patients  Medicare-eligible recipients of skilled home health care | Weight, Height, Weight 6 months ago, calories consumed | 40 | **Nutritional advice**  1. Dietitian intervention to support self-management, incl. goal setting (e.g. to increase calorie intake) plus phone call at weeks 1, 2 and 4, the 60 day FU at home  2. Usual care | None | - Calorie intake - Body weight | 60 days | 3-4* | “The intervention was feasible; however, it did not result in differences between groups for desired outcomes of either caloric intake or body weight.” |
| Nykänen et al. (2014)^39^  Finland  RCT | Patients  Persons who were at risk of malnutrition | MNA | 173 | **Multidisciplinary approach**  1. One comprehensive geriatric intervention with medical intervention (optimisation of medical care), tailored physical activity counselling, and tailored nutritional counselling (1/year) based on MNA score, with nutritionist. The aim was to increase frequency of meals or to add energy. Participants made their own meal plans and received help leaflets. Telephone FU every 2 months.  2. Usual care | None | - MNA - Plasma albinum levels - Body weight - BMI | 2 years | 3-4* | “This study revealed that a nutritional intervention consisting of individual dietary counseling without supplements may lead to an improvement in the nutritional status among home-dwelling older people.” |
| Schilp et al. (2013)^40^  Netherlands  RCT | Patients  Undernourished patients in primary care | Short Nutritional Assessment Questionnaire 65+ (SNAQ 65+) | 146 | **Nutritional advice**  1. Home dietetic intervention by dietitian (who was trained in treating older adults). Intervention included personal action plan, goal setting, motivational interviewing, and workbook. 2. Usual care | Not specified: “additional nutritional supplements” | - Weight, d = 0.13 - physical performance (4 min walk, chair stand, balance), d = 0.03 - hand grip strength, d = 0.02 - daily energy, d = 0.13 and protein intake, d = -0.01 (from food diary) - whole body resistance - fat free mass, d = 0.18 | 3 months and 6 months | 3-4* | “After 6 months, no treatment effect was observed on the primary outcomes body weight, physical performance and handgrip strength, and on the secondary outcomes fat-free mass, energy intake and protein intake.” |
| Trabal et al. (2014)^42^  Spain  Pre-post | Patients  Patients at risk of malnutrition, recently discharged from hospital | MNA | 41 | **Nutritional advice**  Diet enrichment: In a dietary counselling session, patients were asked to add energy and protein rich foods to their main meals (each serving adding 50ckal and 5g protein). Oral information and handout, telephone call after 4 weeks. | None | - Food intake: total energy, protein, carbohydrates and fat intake - BMI - Weight | 4-week and 12-week | 3-4* | The intervention “resulted in increases of both energy and protein intakes. No significant changes were detected in functional status.” |

Notes: BMI = Body Mass Index; MMAT = Mixed Methods Appraisal Tool; MNA = Mini Nutritional Assessment; MNA-SF = Mini Nutritional Assessment-Short from; MOW = Meals on Wheels; MST = Malnutrition Screening Tool; MUST = Malnutrition Universal Screening Tool; ONS = Oral Nutritional Supplements

| *Table S5.* Characteristics of qualitative studies. | | | |  |
| --- | --- | --- | --- | --- |
| Reference, country | Population | Sample size | Aim | MMAT |
| Green et al. (2014)^69^  UK | Healthcare professionals  Nurses (district nurses and community matrons) | 20 | To identify community nurses’ (who care for adults in their own home) barriers and facilitators to undertaking nutritional screening using a screening tool. | 3-4* |
| Hamirudin et al. (2013)^47^  Australia | Healthcare professionals  GPs, general practice registrars and practice nurses | 15 | To identify barriers and facilitators to implementing nutrition screening in older adults among healthcare professionals in the primary care setting. | 0-2* |
|  |  |  |  |  |
| Martin et al. (2014)^70^  Australia | Healthcare professionals  Practice nurses | 181 | To investigate the perceptions of practice nurses on nutrition care for chronic disease management, including specific nutrition-related activities. | 3-4* |
| Moynihan et al. (2012)^48^  UK | Healthcare professionals  Staff involved with providing nutrition | Part 1: 58  Part 2: 22 | To identify the factors that contribute to malnutrition and opportunities for intervention; to design a new system for food provision and nutritional management using an iterative process with ongoing input and feedback from key users; to obtain qualitative evidence on the potential workability and amenability of the new system to become embedded in everyday practice; to investigate the new system's potential application to other important settings for older people, such as residential care homes | 0-2* |
| Dale & Soderhamn, (2015)^49^  Norway | Patients  At risk of malnutrition | 5 | To explore how a small group of older people in Southern Norway perceived their nutritional self-care. | 0-2* |
| den Uijl et al (2014)^71^  Netherlands | Patients  65+ years old, using ONS for treatment of protein energy malnutrition or weight gain purposes, able to give correct answers to two cognitive questions. Some participants were in nursing home; their data is not considered. | 20 | To collect personally relevant factors (product, context, and person factors) that influence ONS consumption | 3-4* |
| Tomstad et al. (2013)^50^  Norway | Patients  Recipients of a home care service | 2 | To evaluate the effects of a study circle by comparing perceived health, sense of coherence, self-care ability, and nutritional risk in two older home-dwelling individuals before, during, and after the study circle, and to describe their experiences of nutritional self-care. | 0-2* |

Notes: ONS = Oral Nutritional Supplements
